# An intervention program based on team building during tactical training tasks to improve team functioning

**DOI:** 10.3389/fpsyg.2023.1065323

**Published:** 2023-03-02

**Authors:** Juan M. Tassi, Miguel A. López-Gajardo, Francisco M. Leo, Jesús Díaz-García, Tomás García-Calvo

**Affiliations:** ^1^Análisis Comportamental de la Actividad Física y el Deporte (ACAFYDE), Departamento de Didáctica de la Expresión Musical, Plástica y Corporal, Facultad de Ciencias del Deporte, Universidad de Extremadura, Av. de la Universidad, s/n, Cáceres, Spain; ^2^Análisis Comportamental de la Actividad Física y el Deporte (ACAFYDE), Departamento de Didáctica de la Expresión Musical, Plástica y Corporal, Facultad de Formación del Profesorado, Universidad de Extremadura, Av. de la Universidad, s/n, Cáceres, Spain

**Keywords:** group cohesion, intra-group conflict, collective efficacy, quasi-experimental design, collective functioning

## Abstract

The study aimed to analyze the effects of an intervention program based on team building developed in technical-tactical training tasks in football. Specifically, it examined the benefits of the intervention in variables related to the conceptual model of team building: role clarity, team identification, intra-team communication, cohesion, intra-group conflict, commitment to the team, inside sacrifice, transactive memory systems, collective efficacy, and perceived performance. The participants were 51 young elite footballers divided into an experimental group (*n* = 27) and a control group (*n* = 24). The methodological design was quasi-experimental with a duration of 8 weeks. The data were collected three times: pretest, posttest, and follow-up. The results showed differences favoring the experimental group compared to the control group after the implementation of the intervention program in the following variables: team identification (*p* < 0.001), role clarity (*p* < 0.001), intra-team communication (*p* < 0.001; except distinctiveness), group cohesion (*p* < 0.05), social conflict (*p* = 0.001), commitment to the team (*p* < 0.001), inside sacrifice (*p* < 0.001), transactive memory systems (*p* < 0.01; except coordination), collective efficacy (*p* = 0.02) and team performance (*p* = 0.001). Consequently, the application of team-building strategies incorporated into specific technical-tactical training tasks in football seems to improve group dynamics in sports teams.

## 1. Introduction

It has been shown that developing group dynamics in sports teams benefits performance in collective sports (Eys et al., 2019). Within this research topic, numerous intervention studies based on team building have been developed (see Eys and Kim, 2017). Team building can be defined as “a method of helping the group to increase efficacy, satisfy its members’ needs, or improve work conditions” (Brawley and Paskevich, 1997, p: 13), which has served as the basis for developing different strategies to such group dynamics like group cohesion or collective efficacy. These strategies have had various characteristics (Eys et al., 2019) but they have rarely been linked to the team’s technical-tactical training, and this may have prevented them from producing the expected efficacy in group variables (Martin et al., 2009). Therefore, Bruner et al. (2013) indicated the need to diversify the type of interventions, increasing the strategies linked to teamwork (McEwan and Beauchamp, 2020). However, they are still not linked to technical-tactical training, which seems essential for the organization of a sports team. Only Leo et al. (2021) proposed strategies based on team building combined with tactical training tasks, obtaining improvements in different psychosocial variables of the team (i.e., task cohesion, intra-group conflict, and collective efficacy). However, to our knowledge, there are no interventions that have tried to integrate team-building strategies during training with defensive and offensive game principles to be carried out by the team within their game system. Therefore, the purpose of this study was to implement an intervention program based on combining team-building strategies and technical-tactical objectives of the game system in training tasks to improve the team’s performance.

### 1.1. Team building in sport

**Figure 1:**
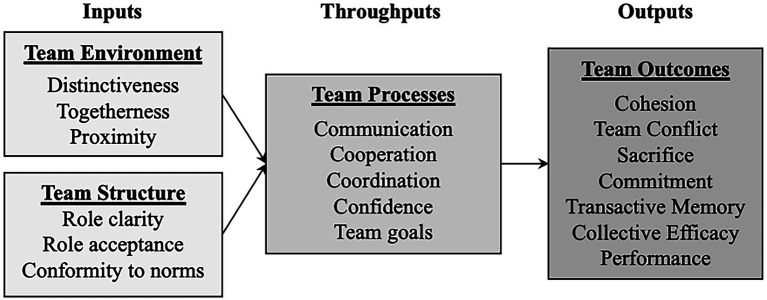
Conceptual model for team building in sport [Adapted From Carron and Spink (1993)].

Carron and Spink (1993) developed a conceptual framework for designing and implementing a team-building program (Figure 1). This linear model consists of inputs, throughputs, and results. This model comprises two inputs: (a) the team environment, which refers to aspects relating to the individual characteristics of group members and the union between them, and (b) the team’s structure, which includes factors such as group roles and norms. Throughputs are a central element and include variables related to team processes such as communication, cooperation, group objectives, etc. Lastly, the team’s outputs refer to the expected results of the proposed intervention (group cohesion, collective efficacy, performance, etc.).

This conceptual framework has served as a reference to develop interventions focused on the construction of sports teams (Martin et al., 2009). These interventions have had various characteristics, with three fundamental elements being considered to obtain positive and stable effects over time (Martin et al., 2009; Bruner et al., 2013). Firstly, the duration of the intervention program appears to be a determining factor (Leo et al., 2009; Martin et al., 2009). Intervention programs that lasted between 2 and 20 weeks showed the most remarkable changes (Martin et al., 2009; Durdubas et al., 2020). In contrast, studies with less than 2 weeks of intervention did not reflect significant effects (Martin et al., 2009). Therefore, team-building-based interventions should establish a prolonged duration to achieve the team’s adaptation and attain the desired results (Martin et al., 2009; Leo et al., 2021).

Secondly, the professional in charge of carrying out the intervention can be a key figure to develop the established strategies optimally. Previous studies have chosen two methods: (a) a direct method, in which the sports psychology specialist works directly with the players, or (b) an indirect method, in which the sport psychology specialist works exclusively with the coach, who is the one who implements the intervention (Carron et al., 1997). Research has shown that both protocols are equally effective but, in many cases, it depends on the characteristics and purpose of the intervention (Martin et al., 2009).

Third, the type of intervention focused on variables of the environment, structure, or processes of the team can also determine the program’s efficacy. Overall, interventions focusing on goal setting (Senécal et al., 2008) or teamwork (McEwan and Beauchamp, 2020) seem to be more effective than other approaches based on extra-sport group activities (Martin et al., 2009). Therefore, it is crucial to reverse the trend of intervention programs that are decontextualized from the team’s game and move towards strategies developed during training, as they seem to be more effective (Leo et al., 2009; McEwan and Beauchamp, 2020; Leo et al., 2021).

Another relevant aspect of team training interventions is the consequences analyzed. In general, improvements have been studied in different measures of performance (Martin et al., 2009) and psychosocial variables like role clarity (Prapavessis et al., 1996), communication (Newin et al., 2008), leadership (Smith and Smoll, 1997) or satisfaction (Carron and Spink, 1993; Bruner and Spink, 2011). However, as some interventions have focused mainly on social aspects, this may be one of the reasons for not-so-significant results (Martin et al., 2009; Bruner and Spink, 2011; Eys et al., 2015). Another reason may be that interventions can influence individual and group psychological processes that have not been assessed. In fact, Bruner et al. (2013) caution that key aspects of other areas of psychology have been largely ignored and that the predominant focus on developing group cohesion in teams can be somewhat restrictive.

### 1.2. The present study

Taking into account the existing scientific literature on the creation of interventions to improve group dynamics, this research seeks to develop an intervention program: (a) intervening on the inputs (i.e., team environment, such as distinction and proximity, and togetherness; team structure, such as role clarity and acceptance, compliance with standards, and leadership); and team processes (i.e., communication, cooperation, coordination, trust, team objectives) of the model of Carron and Spink (1993), integrally, with technical-tactical objectives within the principles of the game, and proposed during football training tasks; (b) It will assess a broad variety of outcome variables, both at the individual level and the team level linked to the model of Carron and Spink (1993), which have shown a close relationship with performance (Beauchamp et al., 2002; Eys et al., 2015; Leo et al., 2022), and will allow the evaluation of the different types of team efficacy (Wang et al., 2014): (1) behavioral processes (i.e., role clarity, intra-group communication, group cohesion, intra-group conflict, transactive memory system (TMS), and collective efficacy); (2) attitudes (i.e., team identification, commitment to the team, and inside sacrifice); and (3) performance (i.e., individual and team performance); (c) developed directly by the sports psychology specialist, establishing sufficient time to obtain significant and stable changes in the group.

Therefore, the main objective of the study was to analyze the effects of an intervention with team-building strategies integrated into training tasks with technical-tactical objectives within the team’s game model to improve group dynamics and team performance. Accordingly, the central hypothesis of the study is that the intervention program developed will favor role clarity, improve team identification, optimize intra-group communication, increase group cohesion and commitment to the team, reduce intra-group conflict, increase teammate inside sacrifice and coordination in game situations, increase the levels of collective efficacy, and improve perceived performance.

## 2. Materials and methods

### 2.1. Design and procedure

To develop the research, we followed the ethical guidelines of the American Psychological Association (2010) and the Declaration of Helsinki (Rickham, 1964). In addition, the present research also received the approval of the Bioethics Committee of the University of the first author (Protocol number: 239/2019). The main researcher contacted the club and coaches of each team to explain the objectives and stages that would be carried out throughout the process. After receiving approval, all participants were informed about the purpose of the study. Next, a consent form was signed by the participant and parent/guardian, guaranteeing participants’ anonymity, confidentiality, and voluntary participation in the research.

Regarding the intervention program, a group of experts in sports psychology and football training was in charge of analyzing, relating, and integrating the technical-tactical objectives of the team (i.e., game principles), with the psychosocial variables of the model of Carron and Spink (1993). This allowed the development of the strategies to be used during the intervention, linked to the different variables that make up the team-building model. In general, these strategies were mainly based on: (a) highlighting aspects of cooperation and organization essential to the game system in each of the training tasks; (b) establishing team values necessary for the improvement of the game model and collective performance; (c) proposing tasks in which players must develop a specific role after assuming and accepting it; (d) allowing players to participate collaboratively in the generation and modification of rules during training tasks; and (e) proposing situations of cooperative training for problem-solving with players’ high interaction and collaboration (see Table 1).

**Table 1:** Intervention program based on team building.

Periods	Contents
1. Introductory stage (2 h)	Meeting to present the objectives, tasks, and dynamics to be developed, as well as the duration of the program.
2. Conceptual stage (2 h)	Meeting to show the theoretical model that supports the intervention, explaining its balance and benefits.
3. Practical stage (2 h)	Meeting where coaches receive detailed training to develop the intervention program.
4. Intervention (8 weeks)	The coaches develop and perform with the players 32 tasks related to the technical-tactical aspects of football during the training sessions on the football field.
A. Team environment	
Distinction and proximity	Develops team identification through the game model and their own training methodology. For example, influence each of the training tasks, the importance of the proper development of their idea of the game. This would allow them to improve collective performance and differentiate them from other teams.
Togetherness	Establish team values, necessary for the improvement of the game model and collective performance. For example, encourage and underline generosity on the part of all players, where each player works for the team in each training task.
B. Team structure	
Role clarity and acceptance	Tasks are proposed in which players must develop a specific role after assuming and accepting it, for example, through tasks where players fulfill a particular function or objective according to their game characteristics or position on the field.
Compliance with norms	Allow players to participate in the generation and modification of norms during training tasks.
Leadership	Generate training situations where players must make decisions autonomously, and shared leadership processes can be developed.
C. Team processes	
Communication	Development of training situations that encourage communication between players, for example, tasks where some players must guide and give directions to their teammates; or, during work breaks, establish players’ meetings to seek solutions or game alternatives.
Cooperation	Propose situations of players’ cooperative training, interaction, and collaboration to solve problems. For example, if everyone collaborates on the task goal, they will score a double goal or two points.
Coordination	Develop training situations where coordination between different players is required to achieve the objective of the task. For example, develop collective attack actions in which players must be coordinated to favor scoring a goal.
Confidence	Strategies that foster players’ individual and collective confidence. For example, develop positive feedback between teammates and the coach to facilitate achieving the goals in training situations.
Team goals	Propose training situations with collective objectives. Achieve or reach something while focusing on team performance. For example, maintaining possession of the ball or progress on the field.

The team-building strategies were carried out in 32 football training tasks (one task each day of the week from Monday to Thursday, for 8 weeks) related to the main objective of each session and associated with the team’s technical-tactical contents (i.e., defensive and offensive). The control and experimental groups both performed the same training sessions, except for the modifications proposed in the specific tasks in the experimental group.

### 2.2. Participants

In the present study, participants were 51 male training footballers belonging to two teams of different categories but the same professional football club of the Argentine first division. Specifically, there were 27 players from the under-17 elite team in the experimental group (*M_age_ =* 16.54*, SD =* 1.23; years of seniority in the club: *M_years_ =* 7.87, *SD* = 1.31), and 24 players from the under-16 elite team in the control group (*M_age_* = 15.44*, SD* = 1.09; years of seniority in the club*: M_years_* = 6.24, *SD* = 1.69). The intervention was carried out with four coaches (i.e., two coaches for each team) between 34 and 42 years old (*M_age_* = 38, *SD* = 3.36), with a coaching license and experience of at least 5 years in training categories. Sample selection was intentional by clusters, considering the geographical proximity of the teams and the working group’s possibilities to access and monitor the sample.

### 2.3. Instruments

#### 2.3.1. Role clarity

To evaluate role clarity, we used the Spanish version validated by Leo et al. (2017) of the Role Ambiguity Scale (RAS: Beauchamp et al., 2002). This instrument has a total of 12 items grouped into three factors: scope of responsibilities and behaviors in fulfilling (i.e., SR&B, six items), evaluation of role performance (i.e., ERP, three items) and consequences of not fulfilling role responsibilities (i.e., RR, three items). An example of role clarity includes “I am clear about the different responsibilities that make up my role.” Players responded to all items on a 9-point Likert scale ranging from 1 (*strongly disagree*) to 9 (*strongly agree*).

#### 2.3.2. Team identification

Team identification was assessed with five items (e.g., “I am very happy to belong to this team”) used in previous research (Fransen et al., 2014; López-Gajardo et al., 2021). Items are scored on a 5-point Likert scale ranging from 1 (*completely disagree*) to 5 (*completely agree*).

#### 2.3.3. Intra-team communication

Intra-team communication was analyzed with the Spanish version of the SECTS-2 (López-Gajardo et al., 2020). This scale begins with the stem phrase “When our team communicates, we..” followed by 15 items (e.g., “we use expressions that only team members understand”) grouped into three factors: acceptance (eight items), negative conflict (four items), and distinction (two items). Players rated all items on a 7-point Likert scale ranging from 1 (*almost never*) to 7 (*almost always*).

#### 2.3.4. Group cohesion

The Spanish version of the Group Environment Questionnaire developed by Leo et al. (2015b) was used. This instrument consists of 12 items (e.g., “Team members join forces to achieve goals during training and matches”) grouped into four factors: Group Integration – Task (IG-T, three items), Group Integration – Social (IG-S, three items), Attractions to the Group – Task (ATG-T, three items), and Attractions to the Group – Social (ATG-S, three items). Items are rated on a 9-point Likert scale, ranging from 1 (*strongly disagree*) to 9 (*strongly agree*).

#### 2.3.5. Intra-group conflict

The Spanish adaptation of the Intra-Group Conflict Scale elaborated by Leo et al. (2015a) was used. This instrument consists of six items (e.g., “Do the team players disagree about the work being done?”), grouped into two factors: social conflict (three items) and task conflict (three items). The items are rated on a 7-point Likert scale ranging from 1 (*never*) to 7 (*always*).

#### 2.3.6. Commitment to the team

To measure players’ perceptions of commitment to the team in the season, we used the Spanish version of the KUT Objective Scale (KUT: Klein et al., 2014). This scale has four items (e.g., “How committed are you to the team?”). Players rated all items on a 5-point Likert scale, ranging from 1 (*strongly disagree*) to 5 (*strongly agree*).

#### 2.3.7. Inside sacrifice

Players’ perceptions of inside sacrifice were measured with the Group Sacrifice Scale (GSS), originally designed by Prapavessis and Carron (1997). This scale has 16 items, drafted positively (e.g., “I’m willing to adopt a play style that does not match my skills for the good of the team”), and grouped into two factors: personal and teammate inside sacrifice. The athletes rated their level of agreement with each statement on a 9-point Likert scale ranging from 1 (*totally disagree*) to 9 (*totally agree*).

#### 2.3.8. Transactive memory system

The Scale of Transactive Memory Systems in Sport (TMMS-S) developed by Leo et al. (2018) was used. This instrument consists of 15 items (e.g., “Each team member is specialized in some aspect of the game”), grouped into three factors: specialization (5 items), credibility (5 items), and coordination (5 items). The response format is a 5-point Likert scale ranging from 1 (*strongly disagree*) to 5 (*strongly agree*).

#### 2.3.9. Collective efficacy

This variable was analyzed with the instrument developed by Leo et al. (2010). This questionnaire presents a stem sentence: “The team’s confidence in our capacity..,” followed by six items that assess the team’s collective efficacy (e.g., “to solve game situations in the attack phase is”). Responses are rated on a 5-point Likert scale ranging from 1 (*bad*) to 5 (*excellent*).

#### 2.3.10. Individual and team performance

Individual and team performance were evaluated through the players’ subjective perceptions. Following the authors Dithurbide et al. (2009), an item was used to measure each type of performance. Thus, players were asked to rate their individual performance (“your individual performance on the team during the season has been..”) and the team’s performance during the season (“the team’s performance during the season has been..”). A 10-point Likert scale ranging from 1 (*poor*) to 10 (*excellent*) was used to evaluate the responses. This way of analyzing team performance seems to be ecologically valid and reliable (Tenenbaum and Gershgoren, 2011), and it has also been used previously by other researchers with football teams (Fransen et al., 2015; Leo et al., 2019; López-Gajardo et al., 2022b).

### 2.4. Data analysis

The data were analyzed with the SPSS 25.0 statistical program. First, means and standard deviations were calculated. Second, the normality of the data was checked with the Shapiro–Wilk test. The data had a normal distribution, so parametric tests were used. Third, one-way analysis of variance (*ANOVA*) was used to test group differences in the dependent variables before starting the study. When significant group differences were observed in the pretest measures, they were included as covariates. Mixed-design *ANOVA*s (3 Times – pretest measures, posttest measures, and follow-up– and 2 Conditions –Experimental and Control) were used to check the effect of the intervention. If this analysis yielded a significant interaction effect, the main effect of time was calculated for each condition. The changes were considered significant when the value of *p* was less than *p* < 0.05.

## 3. Results

Before staring the intervention, there were no significant group differences in SR&B (*p* = 0.133), ERP (*p* = 0.079), personal inside sacrifice (*p* = 0.485), or team performance (*p* = 0.265). On the contrary, However, there were significant pretest group differences in RR (*p* = 0.007), team identification (*p* < 0.001), distinctiveness (*p* < 0.001), positive conflict (*p* < 0.001), acceptance (*p* < 0.001), negative conflict (*p* < 0.001), IG-T (*p* = 0.035), ATG-T (*p* < 0.001), IG-S (*p* = 0.003), ATG-S (*p* < 0.001), social conflict (*p* < 0.001), task conflict (*p* < 0.001), commitment to the team (*p* = 0.001), teammate inside sacrifice (*p* = 0.003), specialization (*p* < 0.001), credibility (*p* < 0.001), coordination (*p* = 0.002), collective efficacy (*p* < 0.001), and individual performance (*p* = 0.007).

Table 2 shows the evolution of SR&B, ERP, RR, and team identification from pretest to posttest and to follow-up between groups. There was a significant Condition x Time interaction for SR&B (*p* < 0.001). Pairwise comparisons revealed that the control group decrease (i.e., impairment) from pretest to follow-up (*p* < 0.001) and from posttest to follow-up (*p* = 0.001). There was also a significant Condition x Time interaction for ERP (*p* < 0.001). Pairwise comparison showed that the control group decrease from pretest to follow-up (*p* < 0.001) and from posttest to follow-up (*p* = 0.001). There was a significant Condition x Time interaction for RR (*p* < 0.001). The experimental group showed an increase in this variable from pretest to follow-up (*p* = 0.009), whereas the control group showed an increase from pretest to posttest (*p* = 0.006) with a decrease from posttest to follow-up (*p* = 0.008). Finally, there was a significant Condition x Time interaction for team identification (*p* < 0.001). The experimental group showed an increase from pretest to posttest (*p* = 0.013) and from pretest to follow-up (*p* = 0.043), whereas the control group showed a decrease from pretest to posttest (*p* = 0.020) and follow-up (*p* < 0.001), and from posttest to follow-up (*p* < 0.001).

**Table 2:** Results of mixed repeated-measures ANOVA in variables related to team structure.

	Control group			Experimental group			Time x condition
	Pre	Post	Follow-up	Pre	Post	Follow-up	*p*
SR&B	8.24 ± 0.40	8.27 ± 0.51	7.70 ± 0.51	8.40 ± 0.37	8.31 ± 0.77	8.43 ± 0.58	<0.001
ERP	8.52 ± 0.73	8.31 ± 0.65	7.68 ± 0.71	8.09 ± 0.94	8.20 ± 0.71	8.24 ± 0.72	<0.001
RR	7.68 ± 1.00	8.26 ± 0.65	7.73 ± 0.52	8.24 ± 0.14	8.40 ± 0.62	8.54 ± 0.49	<0.001
Team identification	4.84 ± 0.14	4.64 ± 0.29	3.82 ± 0.49	4.36 ± 0.52	4.77 ± 0.66	4.63 ± 0.36	<0.001

SR&B = scope of responsibilities and behaviors, ERP = evaluation of role performance; RR = role responsibilities.

Table 3 shows the evolution of distinctiveness, positive conflict, acceptance, and negative conflict from pretest to posttest and follow-up between groups. There was a significant Condition x Time interaction for distinctiveness (*p* = 0.029). Pairwise comparisons showed an increase in this variable from pretest to follow-up (*p* = 0.004) and from posttest to follow-up (*p* = 0.001) in the control group. There was also a significant Condition x Time interaction for positive conflict (*p* < 0.001). The control group showed a decrease from pretest to posttest (*p* = 0.049), from pretest to follow-up (*p* = 0.004), and from posttest to follow-up (*p* < 0.001), whereas the experimental group showed an increase in positive conflict from pretest to posttest (*p* < 0.001) and from pretest to follow-up (*p* = 0.007). There was a significant Condition x Time interaction for acceptance (*p* < 0.001). Pairwise comparison showed decreases in the control group from pretest to follow-up (*p* = 0.004) and from posttest to follow-up (*p* < 0.001), whereas experimental group showed an increase from pretest to follow-up (*p* < 0.001), and from posttest to follow-up (*p* < 0.001). Finally, there was a significant Condition x Time interaction for negative conflict (*p* < 0.001). Pairwise comparison showed decreases in the experimental group from pretest to follow-up (*p* < 0.001) and from posttest to follow-up (*p* < 0.001).

**Table 3:** Results of mixed repeated-measures ANOVA in variables related to team processes.

	Control group			Experimental group			Time x condition
	Pre	Post	Follow-up	Pre	Post	Follow-up	*p*
Distinctiveness	6.38 ± 0.45	6.39 ± 0.54	5.76 ± 0.61	5.21 ± 0.97	5.13 ± 0.91	5.73 ± 0.66	0.029
Positive conflict	6.20 ± 0.55	6.47 ± 0.47	5.81 ± 0.55	4.96 ± 0.42	5.18 ± 0.58	5.75 ± 0.64	<0.001
Acceptance	6.10 ± 0.47	6.33 ± 0.55	5.68 ± 0.45	4.81 ± 0.88	4.74 ± 0.85	5.98 ± 0.48	<0.001
Negative conflict	3.05 ± 0.36	2.98 ± 0.37	3.11 ± 0.42	3.65 ± 0.70	3.67 ± 0.52	2.92 ± 0.49	<0.001

Table 4 shows the evolution of IG-T, ATG-T, IG-S, ATG-S, task conflict, social conflict, commitment to the team, personal and teammate inside sacrifice, TMS factors, collective efficacy, individual performance, and team performance from pretest to posttest and to follow-up between groups. Regarding the cohesion factors, there was a significant Condition x Time interaction for IG-T (*p* = 0.001). Pairwise comparisons showed a significant increase from pretest to posttest (*p* = 0.001) but also a decrease from posttest to follow-up (*p* = <0.001) in the control group, whereas an increase from pretest to follow-up was observed in the experimental group (*p* = 0.005). Also, there was a significant Condition x Time interaction for ATG-T (*p* = 0.005). Pairwise comparisons showed significant decreases from pretest to posttest (*p* < 0.001), from pretest to follow-up (*p* = <0.001), and from posttest to follow-up (*p* < 0.001) in the control group. In contrast, the experimental group showed increases from pretest to posttest (*p* < 0.001), from pretest to follow-up (*p* < 0.001), and from posttest to follow-up (*p* < 0.001). Regarding social cohesion, there was a significant Condition x Time interaction for IG-S (*p* < 0.001). Pairwise comparisons showed decreases from pretest to follow-up (*p < 0*.001) and from posttest to follow-up (*p < 0*.001) in the control group. Likewise, there was a significant Condition x Time interaction for ATG-S (*p* = 0.038). Pairwise comparisons showed that the control group decreased this variable from pretest to follow-up (*p <* 0.001) and from posttest to follow-up (*p < 0*.001), whereas the experimental group showed an increase from pretest to posttest (*p* = 0.048), and also an increase from pretest to follow-up (*p < 0*.001). By contrast, there was no significant Condition x Time interaction for task conflict (*p* = 0.066), but there was a significant interaction for social conflict (*p* = 0.001). Pairwise comparisons showed that the control group increased this social conflict from pretest to follow-up (*p <* 0.001), whereas the experimental group showed decrease from pretest to posttest (*p* = 0.001) and also an increase from posttest to follow-up (*p < 0*.001).

**Table 4:** Results of mixed repeated-measures ANOVA in variables related to outputs – team outcomes.

	Control group			Experimental group			Time x condition
	Pre	Post	Follow-up	Pre	Post	Follow-up	*p*
IG-T	7.37 ± 0.97	8.16 ± 0.55	7.45 ± 0.57	6.62 ± 1.40	7.23 ± 1.08	7.72 ± 0.66	0.001
ATG-T	8.83 ± 0.35	8.36 ± 0.40	7.43 ± 0.78	4.91 ± 1.40	6.96 ± 0.94	8.13 ± 0.66	0.005
IG-S	8.27 ± 0.67	8.48 ± 0.44	7.23 ± 0.77	7.08 ± 1.72	7.46 ± 0.57	7.60 ± 0.86	<0.001
ATG-S	8.68 ± 0.45	8.44 ± 0.57	7.44 ± 0.56	6.76 ± 1.83	7.75 ± 0.99	7.70 ± 0.62	0.038
Task conflict	1.61 ± 1.29	1.98 ± 0.50	2.43 ± 0.45	3.09 ± 1.17	3.22 ± 1.12	3.93 ± 0.84	0.066
Social conflict	1.66 ± 0.84	1.98 ± 0.41	2.33 ± 0.58	3.49 ± 1.48	2.86 ± 1.22	3.74 ± 0.85	0.001
Commitment to the team	5.06 ± 0.55	6.12 ± 0.54	5.49 ± 0.55	4.52±,49	4.57 ± 0.40	5.49 ± 0.71	<0.001
Personal inside sacrifice	8.05 ± 0.43	8.03 ± 0.53	7.03 ± 0.52	8.20 ± 0.97	8.30 ± 0.59	8.40 ± 0.51	<0.001
Teammate inside sacrifice	7.89 ± 0.50	7.90 ± 0.66	6.87 ± 0.63	7.13 ± 1.08	7.59 ± 0.96	8.05 ± 0.72	<0.001
Specialization	4.64 ± 0.15	4.48 ± 0.37	3.75 ± 0.35	3.73 ± 0.44	4.15 ± 0.36	4.45 ± 0.37	0.008
Credibility	3.85 ± 0.29	3.44 ± 0.22	3.76 ± 0.34	6.76 ± 1.83	7.75 ± 0.99	7.70 ± 0.62	<0.001
Coordination	2.65 ± 0.39	3.03 ± 0.24	3.60 ± 0.37	2.27 ± 0.42	2.54 ± 0.41	3.23 ± 0.31	0.535
Collective efficacy	4.52 ± 0.46	4.65 ± 0.34	4.02 ± 0.51	3.19 ± 0.72	3.79 ± 0.55	4.28 ± 0.47	0.021
Individual performance	1.91 ± 0.71	1.91 ± 0.50	1.54 ± 0.50	2.59 ± 0.97	2.66 ± 0.73	2.59 ± 0.50	0.092
Team performance	2.16 ± 0.56	2.16 ± 0.70	1.75 ± 0.73	1.96 ± 0.70	2.66 ± 0.55	2.48 ± 0.57	0.001

IG-T = Group Integration – Task; IG-S = Group Integration – Social; ATG-T = Attractions to the Group – Task, ATG-S = Attractions to the Group – Social.

Analyzing commitment to the team and inside sacrifice, firstly, the results showed a significant Condition × Time interaction for commitment to the team (*p < 0*.001). Pairwise comparisons showed an increase from pretest to posttest (*p < 0*.001) and from pretest to follow-up (*p* = 0.015), but a decrease from posttest to follow-up (*p < 0*.001) in the control group, whereas the experimental group showed increases from pretest to follow-up (*p < 0*.001) and from posttest to follow-up (*p < 0*.001). Secondly, there was also a significant Condition x Time interaction for personal inside sacrifice (*p < 0*.001). Pairwise comparisons showed a decrease in this variable from pretest to posttest (*p < 0*.001) and from pretest to follow-up (*p < 0*.001) in the control group. Thirdly, there was a significant Condition x time Interaction for teammate inside sacrifice (*p < 0*.001). Pairwise comparison showed decreases in the control group from pretest to follow-up (*p < 0*.001) and from posttest to follow-up (*p < 0*.001), whereas increases were observed from pretest to follow-up (*p < 0*.001) and from posttest to follow-up (*p < 0*.001) in the experimental group.

In relation to the TMS factors, there was a significant Condition x Time interaction for specialization (*p* = 0.008). Pairwise comparison showed decreases from pretest to follow-up (*p < 0*.001) and from posttest to follow-up (*p < 0*.001) in the control group, whereas increases were observed from pretest to posttest (*p* = 0.001), from pretest to follow-up (*p < 0*.001), and from posttest to follow-up (*p* = 0.016) in the experimental group. There was a significant Condition x Time interaction for credibility (*p < 0*.001). Pairwise comparison showed a decrease from pretest to follow-up (*p* < 0.01) and an increase from posttest to follow-up (*p* = <0.01) in the control group, whereas increases were observed from pretest to posttest (*p* = 0.001). By contrast, there was not a significant Condition x Time interaction for coordination factor (*p* = 0.535).

Regarding collective efficacy and performance, there was a significant Condition x Time interaction for collective efficacy (*p* = 0.021). Pairwise comparison showed a decrease from posttest to follow-up (*p* = 0.310) in the control group, whereas increases were observed from pretest to posttest (*p* = 0.020) and from posttest to follow-up (*p* = 0.03) in the experimental group. On the contrary, there was no significant Condition x Time interaction for individual performance (*p* = 0.092), but there was a significant Condition x Time interaction for team performance (*p* = 0.001). Pairwise comparison showed a decrease from pretest to follow-up in the control group (*p* = 0.045), whereas increases were observed from pretest to posttest (*p* = 0.003) and from pretest to follow-up (*p* = 0.012) in the experimental group.

## 4. Discussion

The objective of this study was to test the effects of an intervention program with strategies based on team building and integrated into training tasks with technical-tactical objectives and based on the game model to improve the formation of football teams. The results showed differences in most of the psychosocial variables that make up the model of Carron and Spink (1993), between the pretest, posttest, and follow-up measures. In general, the results show the benefits of the team-building intervention program linked to strategies that integrate the team’s defensive and offensive technical-tactical objectives.

Specifically, concerning variables related to the inputs of the team-building model, the dimensions of role clarity and team identification showed a decrease in the control group after the program and at follow-up, whereas the experimental group showed an increase in the posttest (i.e., ERP, RR, and team identification) and at follow-up (SR&B, ERP, and RR). Previous correlational studies have revealed the importance of roles (Beauchamp et al., 2002) and team identification in team performance. However, to our knowledge, there are hardly any studies attempting to improve role clarity and team identification that have measured their improvement (Leo et al., 2021). Therefore, establishing specific roles for the players and informing them of the functions to be performed in the different game situations (defensive and offensive) during the training tasks and the importance of their fulfillment may increase role clarity and commitment (Beauchamp et al., 2002; Leo et al., 2020; Coleman et al., 2021), and, in turn, team identification (Fransen et al., 2014). Therefore, we can state that the intervention program produced benefits in these variables.

Concerning the team processes, the different communication factors presented a similar line of results, with increases in the positive dimensions in the experimental group, mainly at follow-up (i.e., distinctiveness, positive conflict, acceptance), and a decrease in the negative dimension (i.e., negative conflict). On the other hand, the control group showed a decrease in the positive variables and an increase in the negative dimension at follow-up. In this sense, intra-team communication had already been identified as a key process that facilitates a better performance of group members (Leo et al., 2022). Therefore, this study increases previous knowledge, corroborating that the use of tactical tasks with constraints in game situations similar to competitions, where players have to dialogue with their teammates and find collective solutions, seems to improve players’ communicative processes after an intervention, especially in the long term. Setting breaks during work or holding meetings with players to find solutions seem to be optimal strategies to improve player communication in the game.

Concerning the model’s outcomes, in the dimensions of group cohesion and team conflict, the experimental group showed an improvement over the control group in group cohesion but not in team conflict. Two relevant aspects can be extracted from the results: (a) on the one hand, tactical training tasks seem to improve both task and social dimensions in the team, which had not been achieved previously (Leo et al., 2021); (b) on the other hand, intervention programs based on team building do not seem to prevent team conflicts, mainly task conflicts (Leo et al., 2021), perhaps because overcoming conflicts can help to consolidate team cohesion (see Leo and Flores-Cidoncha, 2021).

Continuing with the analysis of the remaining variables, after the intervention, commitment to the team and inside sacrifice presented group differences, with a positive trend in the experimental group compared with the control group. Given the previously found relationship between the two variables with team performance (López-Gajardo et al., 2022a), this study establishes strategies linked to the training tasks and the model that enhance inside sacrifice and commitment to the team.

Concerning the TMS, coordination improved in both groups without differences. The reason for this may be that, as the competition progresses, coordination improves, if only because of the training and matches played by the players. However, the dimensions of specialization and credibility did present differences, with a positive trend in the experimental group compared with the control group. In this sense, it has been proven that the creation and development of shared memory systems (Lewis and Herndon, 2011; Filho et al., 2015), specifically the TMS, are very relevant to improving sports performance (Leo et al., 2018, 2021). Shared memory systems generate high coordination, specialization, and credibility in the team during training, helping to produce play patterns that are very useful in matches (Leo et al., 2018).

Finally, collective efficacy and team performance presented group differences, with a positive trend in the experimental group after the intervention program. Similar results were found by other intervention programs that integrated strategies with tactical training tasks (Leo et al., 2021), as cooperative tasks alone do not appear to be sufficient to improve collective efficacy and team performance (Leo et al., 2009). On the other hand, this tactical training program did not help to improve individual performance, perhaps because of its orientation toward improving the team’s group dynamics. Hence, an individualized technical-tactical training program was not established. Therefore, the design of strategies and tactical constraints aimed at achieving collective objectives improves perceptions of collective efficacy and team performance but not individual performance.

### 4.1. Limitations and future lines of research

Despite the contribution of the study, there are several limitations that must be taken into account for future work. Firstly, the study was conducted in a specific sport with players in training, so caution is recommended when applying some of the strategies in other collective sports or population groups. It would be interesting to analyze other age groups and other collective sports, which could develop team-building strategies associated with tactical training due to their similar characteristics. Secondly, it should be noted that many psychosocial variables linked to the intervention were analyzed, but there was no assessment of the technical-tactical components trained. Therefore, developing an analysis of behaviors related to technical-tactical aspects in training and competitions would be essential. Another limitation of this study is that it used quantitative assessments exclusively, and the involved agents did not assess the intervention program itself through a qualitative approach, which could provide relevant information to perfect the program development. Finally, it should be noted that the number of participants and teams was reduced, so it would be relevant to develop this training program with more sports teams to corroborate the positive results of this intervention.

## 5. Conclusion

The main conclusion of the present study is that the intervention program with strategies based on team-building implemented in the experimental group’s tactical training tasks improved almost all analyzed variables compared with the control group (i.e., role clarity, team identification, intra-team communication, group cohesion, commitment to the team, inside sacrifice, TMS, collective efficacy, and perceived team performance). Therefore, this study provides new knowledge on the development of intervention programs based on team building, mainly the integration of strategies from the model of Carron and Spink (1993) and associated with the defensive and offensive technical-tactical objectives of a football team through specific training tasks. In short, this study can benefit technical corps and sports psychologists who work daily with sports groups similar to those included in this research. In this case, it is recommended to use team-building strategies to simultaneously improve group dynamics and integrate them with the technical-tactical aspects of the team’s game models.

## Data availability statement

The raw data supporting the conclusions of this article will be made available by the authors, without undue reservation.

## Ethics statement

The studies involving human participants were reviewed and approved by Bioethics Committee of the University of the first author (Protocol number: 239/2019). Written informed consent to participate in this study was provided by the participants’ legal guardian/next of kin.

## Author contributions

JT and TG-C designed and performed the study, acquired and interpreted the data, and drafted the first version of the manuscript. JD-G interpreted the data and improved the manuscript. ML-G and FL designed the study, interpreted the data, had overall responsibility for the study, and improved the manuscript. All authors contributed to the article and approved the submitted version.

## Funding

Financial support provided by the European Regional Development Fund (ERDF) and the Government of Extremadura (GR18KA20).

## Conflict of interest

The authors declare that the research was conducted in the absence of any commercial or financial relationships that could be construed as a potential conflict of interest.

## Publisher’s note

All claims expressed in this article are solely those of the authors and do not necessarily represent those of their affiliated organizations, or those of the publisher, the editors and the reviewers. Any product that may be evaluated in this article, or claim that may be made by its manufacturer, is not guaranteed or endorsed by the publisher.
